# Mortality from all cancers and lung, colorectal, breast and prostate cancer by country of birth in England and Wales, 2001–2003

**DOI:** 10.1038/sj.bjc.6603031

**Published:** 2006-03-07

**Authors:** S H Wild, C M Fischbacher, A Brock, C Griffiths, R Bhopal

**Affiliations:** 1 Public Health Sciences, University of Edinburgh, Teviot Place, Edinburgh EH8 9AG, Scotland; 2 Information Services Division (ISD), NHS National Services, Gyle Square, Edinburgh EH12 9EP, Scotland; 3 Mortality Statistics, Office for National Statistics, 1 Drummond Gate, London SW1V 2QQ, UK

**Keywords:** cancer mortality, country of birth, England and Wales

## Abstract

Mortality from all cancers combined and major cancers among men and women aged 20 years and over was compared by country of birth with that of the whole of England and Wales as the reference group. Population data from the 2001 Census and mortality data for 2001–2003 were used to estimate standardised mortality ratios. Data on approximately 399 000 cancer deaths were available, with at least 400 cancer deaths in each of the smaller populations. Statistically significant differences from the reference group included: higher mortality from all cancers combined, lung and colorectal cancer among people born in Scotland and Ireland, lower mortality for all cancers combined, lung, breast and prostate cancer among people born in Bangladesh (except for lung cancer in men), India, Pakistan or China/Hong Kong, lower lung cancer mortality among people born in West Africa or the West Indies, higher breast cancer mortality among women born in West Africa and higher prostate cancer mortality among men born in West Africa or the West Indies. These data may be relevant to causal hypotheses and in relation to health care and cancer prevention.

Cancer mortality in England and Wales has been shown to vary by country of birth in analyses performed around three previous censuses in 1971, 1981 and 1991 (Marmot *et al*, 1984; Balarajan and Bulusu, 1990; Wild and McKeigue, 1997; Gill *et al*, 2002). Information from these studies is useful for health needs assessment, policy formulation, health-care delivery and development of hypotheses regarding the aetiology of disease (Gill *et al*, 2002). A review of the available epidemiological data on cancer in ethnic minority populations up to the mid-1990s concluded that the most common and preventable cancers among minority ethnic populations were the same as those for the general population, but that important differences existed (Bhopal and Rankin, 1996). Patterns of cancer mortality in recent years may have been affected by changing patterns of immigration to England and Wales and increases in the proportion of elderly people in general and migrant populations.

It is not currently possible to examine mortality by ethnicity in England and Wales, as country of birth and not ethnicity is recorded on death certificates. The aim of this analysis was to provide information on cancer mortality for all cancers combined and for the four most common causes of cancer death (lung cancer, female breast cancer, prostate cancer and colorectal cancer) in England and Wales by country of birth for 2001–2003. The analysis of relative mortality for childhood cancers and for less common cancers was beyond the scope of this paper, but important differences by country of birth have been noted in previous studies (Swerdlow *et al*, 1995; Bhopal and Rankin, 1996; Winter *et al*, 1999; Cummins *et al*, 2001).

## SUBJECTS AND METHODS

### Mortality data

Mortality data for the years 2001–2003 by age, sex, country of birth and underlying cause of death were obtained for residents of England and Wales from the Office for National Statistics (ONS). The analysis was restricted to deaths from 2001 in order to use data coded according to the International Classification of Diseases, Tenth Revision (ICD-10) (World Health Organization, 1992) as ICD-9 coding was used until the end of 2000. We included countries, or groups of countries, if there were over 1000 deaths from all causes among adults (⩾20 years of age) born in each country or group of countries between 2001 and 2003. ICD-10 codes were used as follows: all cancers (C00–C97), lung cancer (C33–C34), female breast cancer (C50), prostate cancer (C61) and colorectal cancer (C18–C21).

### Population data

Population data by age, sex and country of birth were obtained from the 2001 Census of England and Wales and are available from the National Statistics website (www.statistics.gov.uk). Place of birth was categorised by individual country (Scotland, India, Bangladesh, Pakistan), the combinations of England and Wales or the Republic of Ireland and Northern Ireland, or country group (East Africa, Eastern Europe, Middle East, North Africa, West Africa, West Indies). The individual countries included in groups of countries are shown in Table 1.

### Statistical analysis

Indirect standardisation was used to adjust for differences in age distribution between populations of interest using the rates in England and Wales in 2001 for the whole population by sex and 5-year age group as the standard. Conventional methods were used to estimate standardised mortality ratios (SMRs) and 95% confidence intervals (CI) by sex and country of birth for people aged 20 years and over (Office of Population Censuses and Surveys and Health and Safety Executive, 1995).

All comparisons in the following text are with mortality in England and Wales as a whole. We regarded results as statistically significant if the 95% CI around SMRs did not include 100. Results are presented in an order defined by geographical groupings.

As people born in England and Wales form a large majority of the study population, their SMRs tend to be close to 100, indicating little difference from the population as a whole. However, as a consequence of the large numbers of deaths among people born in England and Wales, small differences tend to be statistically significant even when they are unlikely to be clinically relevant; no further comment is made on the findings for this group.

## RESULTS

### Populations studied

The total of the subpopulations included in this study represents 99.5% of the adults aged 20 years or older enumerated in the 2001 Census of England and Wales. Age structures differed between subpopulations, with individuals born in Bangladesh and West Africa having a particularly large proportion of people in the 20–44-year age group (data not shown, but available from www.statistics.gov.uk).

### All cancers combined

**Figure 1:**
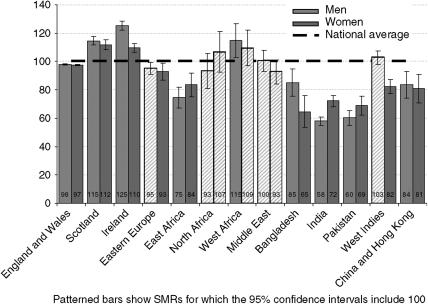
Standardised mortality ratios for all cancer deaths by sex and country of birth, England and Wales, 2001–2003.

There were approximately 399 000 deaths from cancer in the populations studied over the period 2001–2003, of which approximately 365 000 were among people born in England and Wales, 11 200 were among people born in Ireland and 9500 were among people born in Scotland. Standardised mortality ratios for deaths from all cancers for the adult population (aged 20 and over) are shown in Figure 1 (Table 2 gives the numbers of deaths and precise values for CI). Cancer mortality was higher for men and women born in Scotland or Ireland and men born in West Africa, but lower for men and women born in East Africa, Bangladesh, India, Pakistan or China and Hong Kong and for women born in Eastern Europe or the West Indies. It was similar to the whole population for people born in the Middle East, North Africa, for men born in Eastern Europe or the West Indies and for women born in West Africa.

### Lung cancer

**Figure 2:**
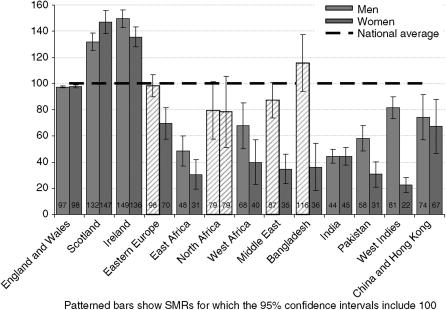
Standardised mortality ratios for lung cancer deaths by sex and country of birth, England and Wales, 2001–2003.

Lung cancer was the commonest specific cancer represented among men (c. 51 000 deaths). There were approximately 33 000 deaths from lung cancer in women, similar to the numbers of deaths from breast cancer, though for women born in Scotland and Ireland the numbers were exceeded by lung cancer. Lung cancer deaths were higher in men than women for all groups, particularly for people born in Bangladesh, the Middle East and the West Indies. Lung cancer mortality was high among men and women born in Scotland and Ireland (see Figure 2 and Table 3), but similar to the reference group among men and women born in North Africa, or men born in Bangladesh, Eastern Europe and the Middle East. For people born in the other countries studied, lung cancer mortality was low.

### Female breast cancer

**Figure 3:**
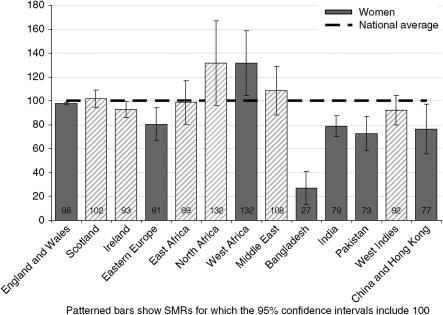
Standardised mortality ratios for female breast cancer deaths by country of birth, England and Wales, 2001–2003.

The overall numbers of deaths among women from breast cancer (33 291) across the study period were similar to those from lung cancer (33 311). For most subgroups by country of birth, with the exception of Scotland and Ireland as mentioned above, the numbers of deaths from breast cancer were either similar (for women born in England and Wales, Eastern Europe or Bangladesh) or exceeded those for lung cancer among women. Breast cancer mortality in women was high among women born in North and West Africa, though the difference was only significant among the latter (see Figure 3 and Table 4). Low SMRs were found in women born in Eastern Europe, Bangladesh, India, Pakistan, China and Hong Kong.

### Prostate cancer

**Figure 4:**
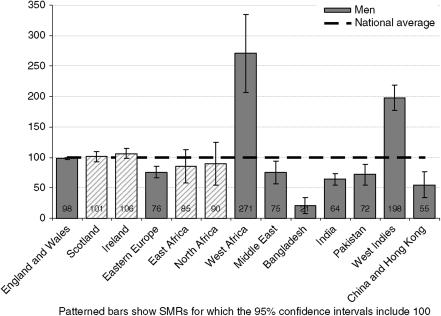
Standardised mortality ratios for prostate cancer deaths by country of birth, England and Wales, 2001–2003.

Mortality from prostate cancer (c. 27 000) was approximately two to three times higher for men born in West Africa and the West Indies (see Figure 4 and Table 5), but low for men born in Eastern Europe, the Middle East, Bangladesh, India, Pakistan, China and Hong Kong.

### Colorectal cancer

**Figure 5:**
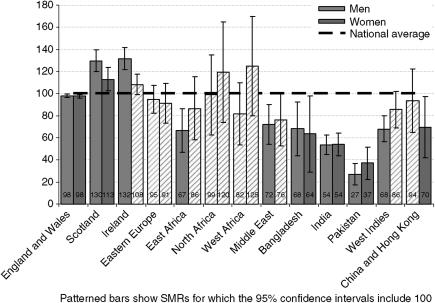
Standardised mortality ratios for colorectal cancer deaths by sex and country of birth, England and Wales, 2001–2003.

There were approximately 22 000 colorectal cancer deaths among men and approximately 20 000 among women; mortality was higher than the national average in men and women born in Scotland, and men born in Ireland (see Figure 5 and Table 6). However, for men and women born in Bangladesh, India and Pakistan, men born in East Africa, the Middle East and the West Indies, and women born in China and Hong Kong, SMRs were significantly lower than for England and Wales as a whole.

## DISCUSSION

This analysis has extended previous work (Wild and McKeigue, 1997) by including a wider range of countries of birth and extending the types of cancer studied, and has shown important differences in cancer mortality by country of birth. A complex combination of genetic and environmental factors, including diet, lifestyle and socio-economic status, contributes to differences in cancer incidence and mortality between populations and is not considered further in this descriptive paper. Migrant studies can be useful in separating the role of genetic and environmental factors (Parkin, 2004). Longer duration of residence in the UK is associated with increased mortality from cancer among South Asians and this may be related to changes in diet and lifestyle (Harding, 2003). Country of birth is not a reliable measure of ethnicity because, for example, a large proportion of people born in East Africa who live in England and Wales are of South Asian ethnicity (see Table S102 of the 2001 Census for England and Wales, available from www.statistics.gov.uk). Further research is required to establish patterns of cancer mortality among offspring of migrants.

Mortality is a function of incidence and survival, and mortality differences may reflect differences in either or both of these factors. In a study of 356 555 cancer cases (ICD-9 140–208) registered between 1990 and 1992 with the Thames, Trent, West Midlands and Yorkshire cancer registries, incidence rates for all cancer sites combined were significantly lower among South Asians than non-South Asians (Winter *et al*, 1999). Data from the Longitudinal Study, based on a 1% sample of the population of England and Wales, have been used to compare cancer incidence among first-generation Scottish, Irish, West Indian and South Asian migrants living in England and Wales (Harding and Rosato, 1999). The high cancer incidence among people born in Scotland or Ireland and low cancer incidence among people born in the West Indies or South Asia is similar to the patterns we have reported for mortality (Harding and Rosato, 1999).

Lung cancer mortality largely reflects smoking habits of populations 20–30 years previously and the higher level in migrants from Scotland and Ireland to England and Wales supports previous findings (Wild and McKeigue, 1997; Harding and Rosato, 1999). Although lung cancer mortality is lower among South Asian populations (other than men born in Bangladesh) than the general population of England and Wales, lung cancer is the commonest cancer among South Asian men, among whom incidence is increasing (Smith *et al*, 2003).

The high breast cancer mortality among women born in North and West Africa (which was only statistically significant in the latter) was unexpected. A nonsignificantly elevated SMR in the 1991 Census for breast cancer among women born in West Africa is unexplained (SMR 125, 95% CI 91–168) (Wild and McKeigue, 1997). A French study that adjusted for confounding factors such as social class and area of residence did not find an excess of breast cancer mortality in migrants from sub-Saharan Africa compared to French natives (Bouchardy *et al*, 1995).

Lower incidence of breast cancer has previously been reported for women born in South Asia and the West Indies (Harding and Rosato, 1999). A national study reported substantially lower breast cancer incidence in South Asians than other women in England and Wales, and found evidence that South Asian women had better breast cancer survival than other women in England and Wales, both overall and in each deprivation category, at least for diagnoses prior to 1990 (Farooq and Coleman, 2005). Better survival in breast cancer among South Asian than in other women in South East England is apparently not explained by differences in age at diagnosis, socioeconomic deprivation or disease stage at presentation (dos Santos Silva *et al*, 2003). However, survival was similar in South Asian and non-Asian women in West Yorkshire, although the former presented later to their GPs, with larger primary tumours, and more frequently underwent mastectomy (Velikova *et al*, 2004).

We found increased mortality from prostate cancer among men born in West Africa or the West Indies, consistent with incidence data which reported a doubling of the two-fold standardised incidence ratios for West Indian men from the Longitudinal Study, compared with all men (2.2, 95% CI 1.0–4.1) (Harding and Rosato, 1999).

Marked differences in the incidence of colorectal cancer have been recorded across the world with the highest rates in North America, Australia and New Zealand and Western and Eastern Europe, and low rates in Africa and Asia (Parkin, 2004). Incidence of colorectal cancer appears to increase rapidly in the first generation of migrants from low- to high-risk areas, suggesting an important aetiological role for environmental factors, particularly diet (McMichael *et al*, 1980). A strong relationship between colorectal cancer and diet is also suggested by the results of ecological studies, which indicate associations between colorectal cancer incidence and intake of meat and fat (Armstrong and Doll, 1975; Prentice and Sheppard, 1990).

Potential sources of bias in these estimates, including errors in population estimates, return migration, numerator–denominator bias (e.g. when country of birth for an individual is recorded differently in the Census and on a death certificate), and inaccurate reporting of cause of death, were previously considered in more detail (Gill *et al*, 2002). Variation may exist in the accuracy of cause of death described on death certificates by country of birth. Under-enumeration in the Census is more marked among some subgroups of the population, such as young men living in inner cities, and this may be even more marked for some further population subgroups. A study in London suggested that current population numbers of older people are underestimated (and therefore SMRs are overestimated), with a particularly marked effect among people born in East Africa, Scotland or Ireland (Bardsley *et al*, 2000).

The issue of misclassification of country of birth among older people who were born in parts of what was previously India that are now Bangladesh or Pakistan does not appear to be a major problem, as mortality ratios for different conditions (e.g. cancer and cardiovascular disease) for people born in India, Pakistan or Bangladesh are not consistently elevated or reduced. Only data for Ireland as a whole could be considered because of the difficulty of separating data for people born in Northern Ireland and the Republic.

The poor uptake of cancer screening among ethnic minorities may mean that cancer mortality in these groups could be reduced if the use of these services could be improved (Caine *et al*, 2001). Data regarding the use of health services among people with cancer from different ethnic groups are limited. A study of the relationship between socio-demographic factors and the components of diagnostic delay (total, patient and primary care, referral and secondary care) for six cancers (breast, colorectal, lung, ovarian, prostate and non-Hodgkin's lymphoma) found that there was greater diagnostic delay for breast cancer for black and South Asian groups (Neal and Allgar, 2005).

## CONCLUSION

The above variations in cancer mortality, which are unlikely to be due to data artefact, may be relevant to causal hypotheses and in relation to health care and cancer prevention.

## Figures and Tables

**Table 1:** List of countries included in groups of countries of birth

**Group**	**Individual countries**
East Africa	Kenya, Malawi, Tanzania, Uganda, Zambia
Eastern Europe	Albania, Belarus, Bosnia and Herzegovinia, Bulgaria, Commonwealth of Russian Independent States nos., Croatia, Czech Republic, Estonia, Hungary, Latvia, Lithuania, Macedonia, Moldova, Montenegro, Poland, Romania, Russia, Serbia, Slovakia, Slovenia, Turkey, Ukraine, Union of Soviet Socialist States nos. and Yugoslavia
Middle East	Armenia, Azerbaijan, Bahrain, Cyprus, Georgia, Iran, Iraq, Israel, Jordan, Kuwait, Lebanon, Middle East nos., Occupied Territories (Gaza and West Bank), Oman, Other Middle East, Qatar, Saudi Arabia, Syrian Arab Republic, United Arab Emirates and Yemen
North Africa	Algeria, Canary Islands, Ceuta and Melilla, Cape Verde, Egypt, Libya, Mauritiania, Morocco, North Africa nos., Sudan and Tunisia
West Africa	Botswana, Gambia, Ghana, Lesotho, Nigeria, Sierra Leone, Swaziland and Zimbabwe
West Indies	Anguilla, Antigua and Barbuda, Bahamas, Barbados, Bermuda, British Virgin Islands, Cayman Islands, Dominica, Gaudeloupe, Grenada, Jamaica, Martinique, Montserrat, Netherland Antilles, St Christopher (St Kitts) Nevis, St Lucia, St Vincent and the Grenadines, Trinidad and Tobago, Turks and Caicos Islands, US Virgin Islands, West Indies, West Indies nos.

**Table 2:** Numbers of deaths and all cancer (ICD-10 C00–C97) SMRs by sex and country of birth for people aged 20 years and above

	**Men**		**Women**	
	**No. of deaths**	**SMR (95% CI)**	**No. of deaths**	**SMR (95% CI)**
England and Wales	188 495	98 (97–98)	175 976	97 (97–98)
Scotland	5271	115 (111–118)	4372	112 (109–115)
Ireland	6110	125 (122–129)	5130	110 (107–113)
Eastern Europe	1979	95 (91–100)	878	93 (87–99)
East Africa	384	75 (67–83)	361	84 (75–93)
North Africa	206	93 (81–108)	197	107 (92–104)
West Africa	352	115 (103–128)	280	109 (97–124)
Middle East	685	100 (93–109)	447	93 (84–102)
Bangladesh	283	85 (75–96)	117	65 (53–79)
India	1440	58 (55–61)	1410	72 (68–76)
Pakistan	526	60 (55–66)	414	69 (62–76)
West Indies	1679	103 (98–108)	996	82 (77–88)
China and Hong Kong	287	84 (74–94)	240	81 (71–93)

CI=confidence interval; ICD=International Classification of Diseases; SMR=standardised mortality ratio.

**Table 3:** Numbers of deaths and lung cancer (ICD-10 C33–C34) SMRs by sex and country of birth for people 20+ years of age

	**Men**		**Women**	
	**No. of deaths**	**SMR (95% CI)**	**No. of deaths**	**SMR (95% CI)**
England and Wales	46 280	97 (97–98)	30 710	98 (97–99)
Scotland	1506	132 (125–139)	1026	147 (138–157)
Ireland	1848	149 (143–157)	1167	136 (128–144)
Eastern Europe	497	98 (90–108)	118	70 (57–84)
East Africa	61	48 (37–62)	22	31 (19–46)
North Africa	43	79 (58–107)	26	79 (51–115)
West Africa	50	68 (50–89)	16	40 (23–65)
Middle East	148	87 (74–103)	30	35 (23–50)
Bangladesh	99	116 (94–141)	11	36 (18–65)
India	279	44 (39–50)	158	45 (38–53)
Pakistan	128	58 (48–70)	32	31 (21–43)
West Indies	348	81 (73–91)	51	22 (17–29)
China and Hong Kong	63	74 (57–95)	34	67 (47–94)

CI=confidence interval; ICD=International Classification of Diseases; SMR=standardised mortality ratio.

**Table 4:** Numbers of deaths and breast cancer (ICD-10 C50) SMRs among women of 20+ years of age by country of birth

	**No. of deaths**	**SMR (95% CI)**
England and Wales	30 759	98 (97–99)
Scotland	700	102 (94–110)
Ireland	736	93 (86–100)
Eastern Europe	126	81 (67–97)
East Africa	101	99 (80–122)
North Africa	45	132 (96–176)
West Africa	83	132 (105–163)
Middle East	102	108 (88–133)
Bangladesh	11	27 (14–49)
India	284	79 (70–89)
Pakistan	91	73 (58–91)
West Indies	208	92 (80–106)
China and Hong Kong	45	77 (56–103)

CI=confidence interval; ICD=International Classification of Diseases; SMR=standardised mortality ratio.

**Table 5:** Numbers of deaths and prostate cancer (ICD-10 C61) SMRs among men of 20+ years of age by country of birth

	**No. of deaths**	**SMR (95% CI)**
England & Wales	24 322	98 (97–99)
Scotland	579	101 (93–110)
Ireland	638	106 (98–115)
Eastern Europe	247	76 (67–87)
East Africa	31	85 (58–121)
North Africa	20	90 (55–139)
West Africa	60	271 (207–349)
Middle East	53	75 (57–99)
Bangladesh	6	21 (8–45)
India	183	64 (55–75)
Pakistan	61	72 (55–93)
West Indies	350	198 (178–221)
China and Hong Kong	20	55 (34–85)

CI=confidence interval; ICD=International Classification of Diseases; SMR=standardised mortality ratio.

**Table 6:** Numbers of deaths and colorectal cancer (ICD-10 C18–C20) SMRs by sex and country of birth for people 20+ years of age

	**Men**		**Women**	
	**No. of deaths**	**SMR (95% CI)**	**No. of deaths**	**SMR (95% CI)**
England and Wales	20 084	98 (97–99)	18 099	98 (96–99)
Scotland	635	130 (120–141)	442	113 (103–125)
Ireland	685	132 (122–142)	506	108 (99–119)
Eastern Europe	208	95 (82–110)	89	91 (73–112)
East Africa	36	67 (47–92)	29	86 (58–124)
North Africa	23	99 (63–148)	21	120 (74–183)
West Africa	26	82 (53–120)	24	125 (80–186)
Middle East	52	72 (54–94)	33	76 (53–107)
Bangladesh	24	68 (44–101)	9	64 (29–121)
India	142	54 (45–64)	100	54 (44–66)
Pakistan	25	27 (18–40)	19	37 (22–58)
West Indies	119	68 (56–82)	93	86 (69–105)
China and Hong Kong	34	94 (65–131)	19	70 (42–109)

CI=confidence interval; ICD=International Classification of Diseases; SMR=standardised mortality ratio.
