# Mortality from all cancers and lung, colorectal, breast and prostate cancer by country of birth in England and Wales, 2001–2003

**DOI:** 10.1038/sj.bjc.6603263

**Published:** 2006-08-01

**Authors:** S H Wild, C M Fischbacher, A Brock, C Griffiths, R Bhopal

**Correction to**: *British Journal of Cancer* (2006) **94**, 1079–1085. doi:10.1038/sj.bjc.6603031

Owing to an author error, the data presented in Figure 5 were incorrect. The correct data are presented in a revised Figure 5, shown below:

## Figures and Tables

**Figure 5:**
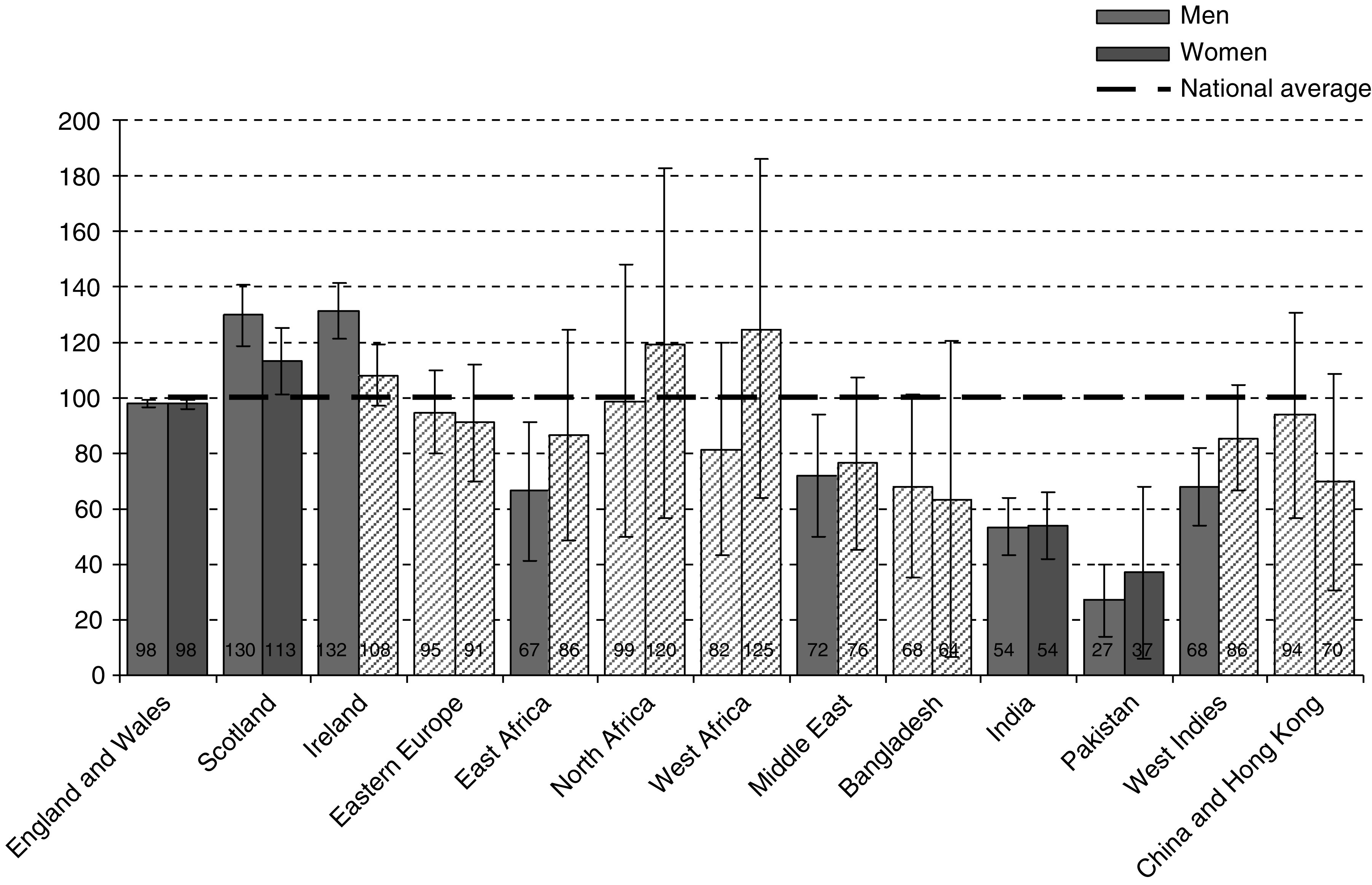
SMRs for colorectal cancer deaths by sex and country of birth, England & Wales, 2001–2003. Solid bars indicate SMRs for which 95% confidence intervals exclude 100.

